# Exchanging Information About Reproductive and Sexual Health on the US-Mexico Border: A Good Example of Collaboration

**Published:** 2008-09-15

**Authors:** Patricia Uribe Zúñiga

**Affiliations:** National Center for Gender Equity and Reproductive Health, Under-Secretariat of Health Promotion and Prevention, Secretariat of Health

Public health is a human right that transcends borders yet is defined by socioeconomic, demographic, and cultural differences. In other words, it is connected to a nation's development. Mexico and the United States share a 3,326-km border that includes 4 US and 6 Mexican border states, more than 20 border crossing points, and an intensive economic, cultural, and commercial exchange (1). Concerted binational efforts are necessary to preserve and maintain the best possible conditions to protect the health of our citizens.

The ability to share technical information about sexual and reproductive health, especially in the area of maternal and child health, in key locations on our shared border creates opportunities to exchange experiences and improve the epidemiologic profile of our binational border residents. These opportunities arise through assimilating and adopting successful experiences and learning from occasional mistakes and documented obstacles to program operations.

The US-Mexico border region is an area of marked demographic, social, and cultural differences, a transition zone between a developed country and another that is still developing, characterized by distinct health profiles. The mosaic of public health issues and the demographic, epidemiologic, and socioeconomic dynamics that need to be addressed in this region are broad. Reproductive and sexual health issues are of special interest, and addressing them is a priority, given the nature of risk factors, the complexity of the approaches needed, and the high cost of consequences.

On the Mexican side of the border, the high birth rate coupled with poverty and migration result in operational problems in the area of sexual and reproductive health care. This situation requires preventive interventions for both countries, taking into consideration their existing economic, political, and cultural differences.

The Brownsville-Matamoros Sister City Project for Women's Health (BMSCP) emerged from this need. The project was conceptualized considering the 14 pairs of sister cities, the 44 US border counties, and the 80 Mexican municipalities in the region. The objective was to promote strategies for prevention and health protection on the border. Communities in the region share many characteristics, including a constant migratory influx that necessarily carries a number of social determinants that negatively affect health, particularly reproductive and sexual health.

The government of Mexico has developed a strong maternal and perinatal health policy through the implementation of the program "Arranque Parejo en la Vida" ("Fair Beginning of Life"). This program uses the best evidence-based practices to increase maternal and perinatal health and has proved to be effective locally. Program strategies include professional development for the health care workforce, strong stimuli to engage community participation, and creation of a network of health care and social support in maternal and perinatal health. Furthermore, immediate response to all reported maternal deaths allows us to obtain detailed information for each contributing cause of death, enabling evidence-based decisions to prevent future deaths associated with similar risk factors.

Mexican public health policy places careful attention on the perinatal period to prevent congenital and other disabilities that have affected maternal and perinatal health. Mexico's crusade to avert congenital disabilities began with interventions such as the promotion of breastfeeding, folic acid supplementation, neonatal screening, and neonatal resuscitation.

The National Center for Gender Equity and Reproductive Health is responsible for implementing public policies for the prevention, control, and treatment of uterine-cervical cancer and for conducting national family planning and contraceptive care programs. These programs are assisted by specific action plans, which combine evidence-based practices and innovative strategies that have proved their effectiveness at the local level.

This issue of *Preventing Chronic Disease* *(PCD) *presents the results of the BMSCP in a set of scientific reports that reflect existing health problems and the beneficial results of effective interventions. One example is the promotion of exclusive breastfeeding, which brings immediate and long-term benefits to the health of mothers and children. This health policy has brought tremendous advances to Mexico, promoting national standards that regulate the practices of infant formula manufacturers to prevent them from interfering with natural forces that promote breastfeeding. Nevertheless, we need to enhance strategies to increase the number of newborns who are breastfed. According to the National Health and Nutrition Survey of 2006, the level of breastfeeding is slightly more than 90% in Mexico, with a mean duration of 5.5 months nationwide (2). We shall continue advancing on this topic, securing favorable workplace conditions for working mothers and increasing awareness and support from men.

The study also drew attention to prenatal testing for human immunodeficiency virus (HIV), which simply and cost-effectively allows timely detection and treatment of HIV infection in pregnant women and possible prevention of perinatal HIV infection. The number of HIV infections associated with perinatal transmission should be lower in Mexico, given the fact that Mexico has secured free access to antiretroviral therapies for anyone who needs them. Pregnant women who lack health insurance have increased access to free HIV screening tests. Two obstacles to preventing perinatal HIV transmission in Mexico are the low perception of risk among women and the low coverage of the HIV screening test because of a policy that recommended screening only for pregnant women who had had a positive screening test result for syphilis. (The policy was changed in 2008.)

At the time of the BMSCP (2005), when HIV prenatal testing rates in Matamoros, Mexico, and Cameron County, Texas, were compared, Mexico did not yet have a universal policy for HIV screening and antiretroviral therapy. As a result, a solid and articulated strategy to prevent mother-to-infant HIV transmission was difficult. Now that Mexico has changed its policy, we need to address cultural and other factors that may affect awareness and behavior of health personnel and impede timely HIV testing of all pregnant women. Lessons may be learned from the successful experience in Cameron County.

The mortality rate associated with cervical cancer in Mexico is 2.5 times higher than that of Hispanic women living in the United States (3,4). The BMSCP estimated that Mexican women aged 25 years or younger had a lower prevalence of Papanicolaou testing. This finding is consistent with the Official Mexican Norm, which promotes detection among women aged 25 years or older, also in agreement with the recommendation of the World Health Organization (5). The proportion of women who have been tested varies from 43.7% for women aged 20 to 24 years to 73.1% for women aged 25 to 29 years (6).

Given the vision of the National Center for Gender Equity and Reproductive Health, I am certain that this exchange of information will enrich both countries because it will create ways to generate new health strategies and to improve existing ones. I am grateful for the opportunity to contribute to this project and believe that this surveillance activity will continue and expand to other borders.
